# PAmiRDB: A web resource for plant miRNAs targeting viruses

**DOI:** 10.1038/s41598-019-41027-1

**Published:** 2019-03-15

**Authors:** Deepshikha Satish, Sunil Kumar Mukherjee, Dinesh Gupta

**Affiliations:** 1 Translational Bioinformatics Group, International Centre For Genetic Engineering and Biotechnology, New Delhi, 110067 India; 2 Division of Plant Pathology, Indian Agricultural Research Institute, New Delhi, India

## Abstract

MicroRNAs (miRNAs) have emerged to be essential constituents of host antiviral-defense mechanisms. The miRNA mediated antiviral mechanism was first experimentally established in animals, which proved that host miRNAs regulate viral gene expression by targeting the animal virus mRNAs. There are comparatively fewer reports about such interactions in plants, however, artificial miRNA studies prove that miRNAs play similar antiviral role in plants too. To explore the extent of this phenomenon in plant genomes, and in the absence of any publicly available resource for prediction of plant miRNAs targeting viruses, we were motivated to predict such interactions of plant miRNAs and viral genes. The intriguing results of the predictions are compiled as a database, which we have named as PAmiRDB. The current version of PAmiRDB includes more than 2600 plant miRNAs and their specific interactions with corresponding targets in approximately 500 viral species (predominantly from the major plant-infecting virus families of geminiviruses and potyviruses). PAmiRDB is a database of known plant miRNAs and their predicted targets in virus genomes. The innovative database query-interface enables global and comprehensive investigation of such predicted interactions between host miRNAs and viral genes. The database integrated-tools also helps researchers to design experiments to confirm such interactions. PAmiRDB is available at http://bioinfo.icgeb.res.in/pamirdb

## Introduction

Plant viruses are the major hurdles in realizing the United Nation’s sustainable development goal of zero hunger. The menace of virus epidemic causes huge economic losses to agriculture industries all over the world. Geminivirus and Potyvirus families are majorly responsible for the virus mediated devastation of crops, affecting food security, especially in South-East Asia and Africa^1,2^. Geminiviruses have single-stranded circular DNA genomes, which are vectored by white-flies, whereas Potyviruses are RNA viruses. These two virus families account for nearly 40 percent of the currently known plant viruses. Being strict intracellular pathogens, viruses cannot be effectively controlled using chemical agents. Prophylactic measures to control vector population mainly involves destruction of infected plants and use of excessive pesticide applications. The harmful effects of these pesticides in the environment are conspicuously detrimental, hence considerable efforts are being made to devise sustainable organic farming. These facts motivate researchers to discover the intracellular host genetic defense mechanisms against the viruses which can help in the development of novel, permanent, and long-lasting solutions against the viruses.

During the course of evolution, as a survival measure, the plants have developed complicated mechanisms to resist viral infections. One of the mechanisms which has drawn considerable interest amongst researchers is RNA silencing. Plants and other eukaryotes use RNA silencing to protect their genomes against aberrant nucleic acids and viruses^3,4^. RNA silencing is best illustrated by RNAi using short RNAs (20–30 nt) which manipulate complementary nucleic acids with the help of host RNAi factors. The short RNAs can be classified into five classes of small regulatory RNAs (miRNAs, siRNAs, ta-siRNAs, nat-siRNAs, and Piwi-interacting RNAs). Amongst these, the miRNAs are small endogenous non-coding RNAs of approximately 22 nucleotide length which are very crucial regulators of gene expression, in disease as well as normal regulatory processes in eukaryotes.

Various artificial miRNA probes reiterate the role of miRNAs in viral defense in plants^5–14^. However, the interplay between viruses and RNA interference machinery in animals as well as plants is complicated by the fact that viruses may also counter or use RNAi for their own replication using virus-encoded RNA silencing suppressors^15–17^. Along with this, the viral encoded RNA silencing suppressor proteins too interfere with host RNA machinery, to facilitate viral infection by evading host immunity^15^. These pieces of evidences make it clear that interactions among plant miRNAs and viral genomes are very complex, and hence requires further theoretical as well as experimental investigations.

The experimental investigations are hard to perform and often fail to provide clear indications of such interactions due to technical limitations. Hence, bioinformatics aided predictions of miRNA and its corresponding targets may provide useful insights into the phenomenon and play an important role in guiding such experiments.

The availability of several plant and virus genomes provides unique opportunity to generate comprehensive information related to the interaction of plant miRNAs with Geminivirus and Potyvirus target genes. In the present study, we have developed ‘PAmiRDB’ (Plant antiviral miRNA database), with an aim to provide genome-wide predictions related to site-specific viral genome interactions with plant miRNAs through a single web-based publicly available resource. Apart from sequence-based alignment of miRNA-target interaction, PAmiRDB also provides an insight into interaction sites on various viral genomes i.e. miRNA target sites and various virus proteins and free energy of the miRNA-target pairing. PAmiRDB is freely accessible at http://bioinfo.icgeb.res.in/pamirdb. PAmiRDB also provides additional information about plant viruses, their hosts, and predicted target proteins, through dynamically generated links, retrievable from the publicly available repositories.

The current version of PAmiRDB also contains information about the miRNAs encoded by the two most important plant virus families, namely, Geminiviridae and Potyviridae. Since it has been observed that different viruses have specific critical site pairing^18^, the site-specific tweaking search options in PAmiRDB makes the database immensely useful to retrieve most probable predicted antiviral miRNAs. The database provides researchers with a useful initiation point to design experiments aimed towards the discovery of antiviral miRNAs and mechanisms of host miRNA networks. Though now we have genomic information about a number of plant species, we have focused on predictions of miRNAs encoded by seven major plant species (*A. thaliana, G. max, O. sativa, S. bicolor, S. licopersicum, V. vinifera, Z. Mays*), which are also known to be infected by some of the most debilitating viral infections with wide global economic impact. Other reasons for the choice of the seven plant species are technical as each of the species has more than least 600 experimentally validated miRNAs, and are hosts for at least ten fully sequenced plant-infecting viruses.

### Web portal and tools

**Figure 1:**
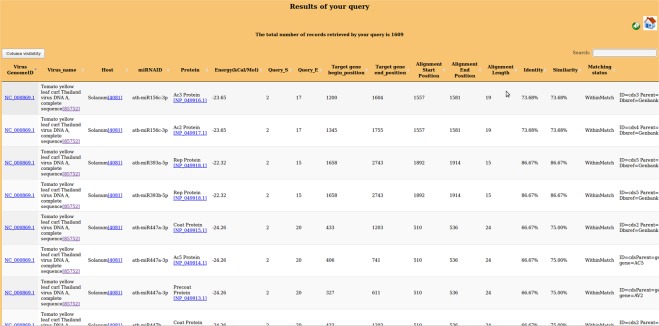
Result Page of PamiRDB. This is a dynamic page which gives user option to sort the results according to the column of interest. Search column allows finding the desired result on the page exclusively.

The user-friendly query interface of PAmiRDB provides query options to specify a virus family, compulsory binding sites in a miRNA-target pair, specific plant species, other options for query cover, percentage identity and energy threshold. Alternately, more specific results may be obtained quickly by using the drop-down menu for user-defined queries to include either genome ID, virus name, target protein and maximum energy. PAmiRDB accepts queries searching complementarity between a list of miRNA IDs with either all the proteins of a user-defined virus or a specific protein of all the viruses of geminivirus/potyvirus family. The feature makes PAmiRDB immensely useful for the researchers interested in a few miRNAs of interest. PAmiRDB results (Fig. 1) also consist of links to the alignments of individual results.

Apart from miRNA pairing with its target, the position of miRNA alignment on virus genome is also crucial in deciding the effectiveness of binding and hence gene regulation^9^. To address this issue PAmiRDB gives insight regarding the location of miRNA binding on viral genomes. It is worth mentioning here that widely adopted rules in artificial microRNA (amiRNA) design describe position 9–11 to play a vital role in miRNA-mediated gene silencing, and mismatches at positions 3–6, 9 and 12 are more critical (failure of amiRNA-mediated resistance), positions 2, 10, 11, 13, 15 and 18 are of intermediate importance, while the remaining positions are least critical for RNA silencing^19^. Thus, by using PAmiRDB, one may retrieve results by specifying particular positions for which binding is necessary.

## Results and Discussion

In the PAmiRDB output pages (Fig. 1), the virus Genome IDs are linked to relevant information of miRNA-target interactions involving the corresponding plant miRNA and its targets within the complete viral genome. Further, the results page enlists the alignments with the viral genome to give an idea about the accessibility of a gene to miRNA, and whether the miRNA lies completely within the gene or pairs with the flanking region of aligned target gene. The results page also gives an option to sort results according to various parameters, including energy and host virus name (in alphabetical order).

Details regarding the viruses can also be retrieved along with its predicted targeted proteins and the corresponding miRNAs. This can help in the analysis of protein specific miRNA targets e.g. resistance involving Coat Protein. Additionally, taxonomic details of the host plant can be retrieved using corresponding hyperlinks.

**Figure 2:**
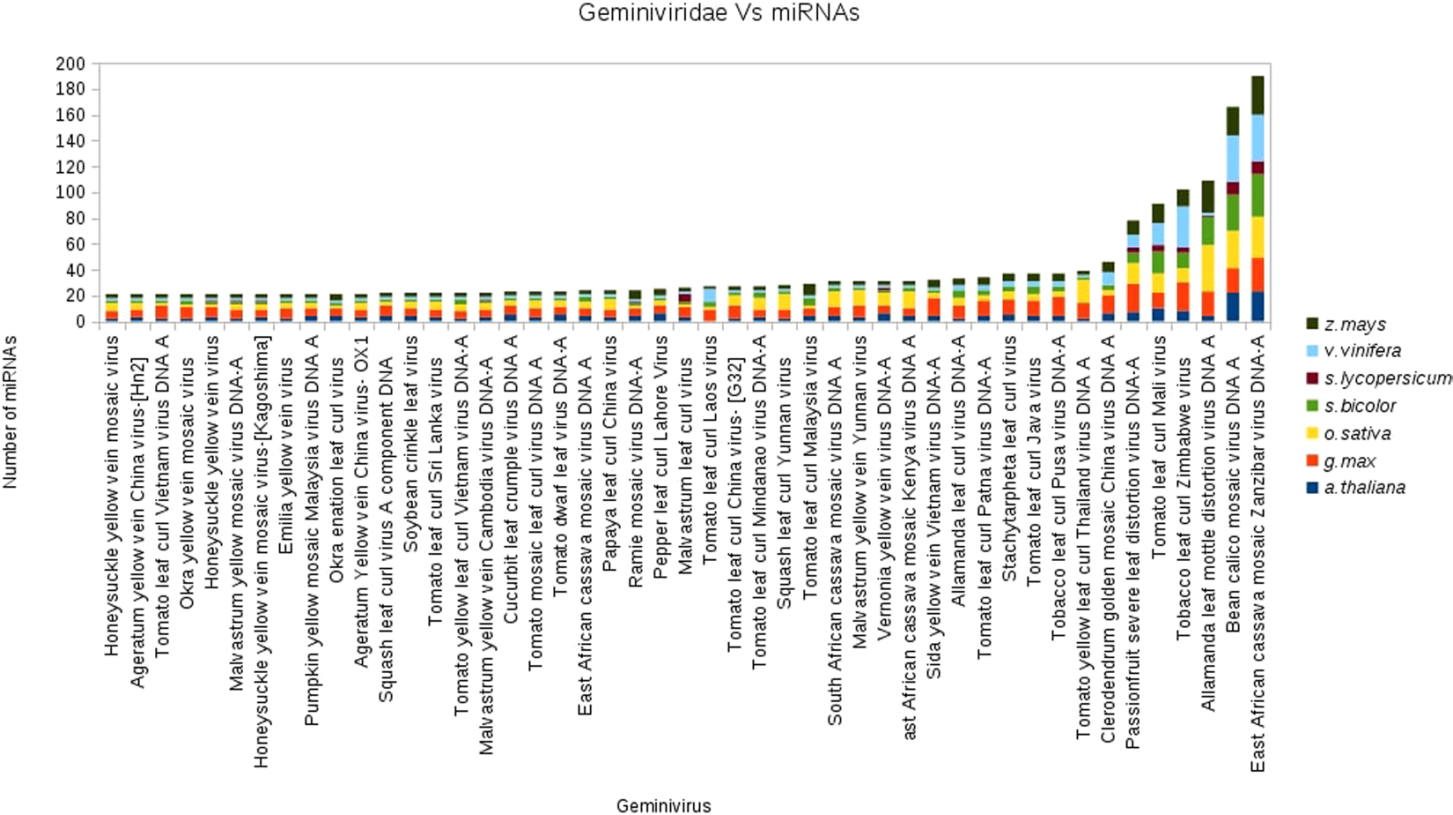
Geminivirus Vs number of miRNA targets found. This graph depicts data of 7 plant species: *A. thaliana, G. max, O. sativa, S. bicolour, S. lycopersicum, V. vinifera, Z. mays*. Viruses with more than 20 complimentary miRNA only, have been shown in the figure.

A deeper analysis of miRNA-gene pairs indicates interesting observations- for example, few miRNAs have more complimentary pairs than others (Fig. 2). Several miRNAs also have complementary pairs even in those viruses, which are not the natural host of the plants. This indicates that during the course of evolution, resistance against few viruses became an integral part of plant genomes to impart immunity against viruses. We speculate that such molecular interactions may be one of the factors conferring natural resistance of few plants against specific viruses.

In plants, the complementarity between miRNA and its potential target site determines the stability of miRNA–mRNA duplex and efficacy of target site which has been utilized as a key selection criterion for target gene analysis^20,21^. The 5′ regions of plant miRNAs are most complementary to target mRNAs^22^. Complementarity to a miRNA seed region is sufficient to induce moderate repression of a target transcript in the unicellular green alga and animals. On the other hand, in plants, the seed region pairing is sometimes not an absolute necessity for being a match^23,24^. However, it has been established that a single mismatch at the 5′ end of miRNA significantly diminishes target site efficacy, and two or more consecutive mismatches at the miRNA 5′ fully abolish it^21^. Keeping in view the characteristics of seed regions, PAmiRDB shows only the miRNA-mRNA pairs with perfect complementarity in seed regions.

Plant miRNAs have evolved to optimize cleavage efficiency because of their high complementarity to their targets^25^. Three or more mismatches are permitted between miRNA and its target, which thereby significantly expands the spectrum of targets and facilitates the release of cleaved target RNAs from the RNA Induced Silencing Complex (RISC)^16^. Complementarity between miRNA-mRNA leads to stability of interaction between the pairs and thus lowers the free energy. PAmiRDB shows only the results with interaction energy up to −20 Kcal/mol, thus providing more reliable and true positive results.

Earliest studies in Arabidopsis indicate, near-perfect complementarity between the miRNAs and their targets as a general rule of antiviral activities, however, subsequent research indicated that pairing at some sites may be less perfect than others. Apart from this observation, residues at few specific positions are found to be more crucial than others, for instance, position 19 of miR319 and position 16 in the target region in mRNAs were shown to be critical for pairing in Arabidopsis^26^. Likewise, a mismatch at the 10th and 11th miRNA positions could lead to inhibition of translation, instead of target cleavage^27^. As different viruses have different critical sites^10^, the site-specific query option makes PAmiRDB quite useful for query-based retrieval of antiviral miRNAs.

## Conclusion

PAmiRDB is the first-ever comprehensive database of predicted plant miRNA interactions with virus genomes. PAmiRDB is a useful tool to investigate the interactions between host plant-miRNAs and commonly plant-infecting virus genes. It is useful to narrow down a list of predicted and most likely interacting miRNAs-mRNA pairs, which may be experimentally verified using experimental tools like artificial miRNA technology or generating recombinant viral genomes. The web-based PAmiRDB interface provides queries to search specific virus, miRNA or target protein by using the keywords free energy threshold, query coverage, identity percentage, and interaction site restriction. The unique feature of customized queries makes the database more useful to quickly retrieve a list of most probable targets in plant infecting viral genomes. The results contain relevant details of miRNA-target alignments, the proteins coded by predicted target genes, making the results more useful for further investigation.

The PAmiRDB database will be developed further to include more plant miRNAs and their interactomes involving viral genomes. We will continuously update and develop PAmiRDB to enhance its data content and functionality. The major data content enhancement will come from the elaboration of the gene annotation, incorporation of additional miRNAs from other species with fully annotated genomes, and incorporation of the evolutionary conservation status of miRNAs involved in such interactions.

## Methods

**Figure 3:**
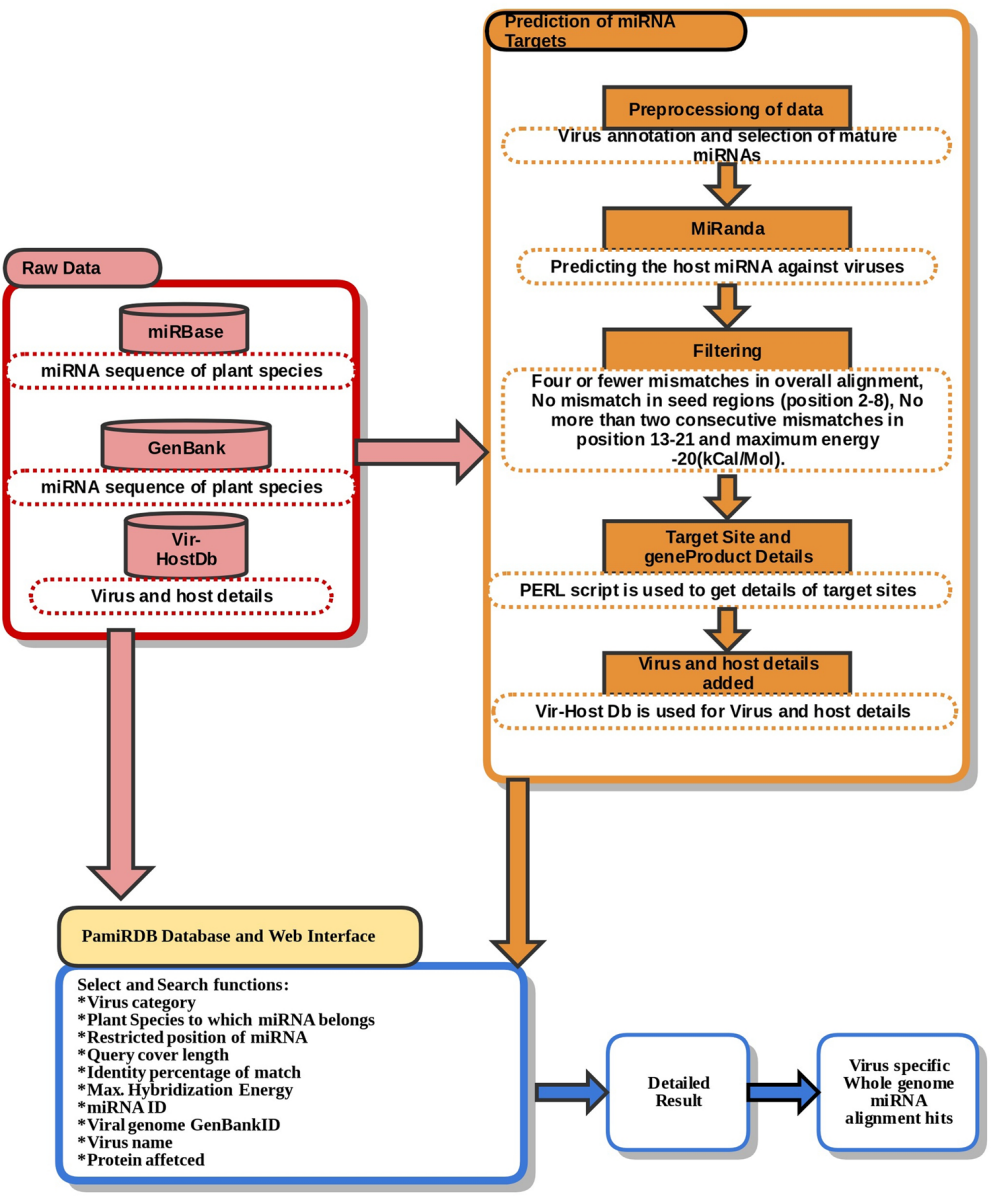
The flowchart depicts the schema followed during database generation.

PAmiRDB development work-flow is shown in Fig. 3. The miRNAs sequences wand virus genomes were retrieved from miRBASE^28^ and Genbank^29^, respectively. Host-ranges, taxonomic details of plant viruses and hosts were obtained from Virus-Host Db^30^.

We modified and customized miRanda tool to filter out the most true positive miRNA-gene pairs^31^. We used the criteria to output the pairs with no mismatch in seed regions (position 2–8), four or fewer mismatches in overall alignment, no more than two consecutive mismatches in position 13–21, a maximum interaction energy of −20 (kcal/Mol), and used it for target search for miRNAs in virus genomes. The original miRanda tool provides elaborate results and it is challenging to parse position specific outcomes. Hence, PamiRDB will also be useful to the biologists not trained in bioinformatics.

Bioinformatics investigations into the antiviral attributes of plant miRNAs using the above-mentioned criteria revealed that a significant number of plant miRNAs preferentially target genomes of phytopathogenic viruses, as compared to the negative controls, which includes randomly generated miRNAs or genomes of animal viruses^32,33^.

Despite the fact that miRanda is designed for prediction of miRNA targets in animal genomes, it can be successfully used for the prediction of miRNA targets in viruses and plants^34^. In miRanda, a dynamic programming based local alignment is carried out between the query miRNA sequence and the reference sequence. The software uses a scoring system based on the complementarities of nucleotides, similar to the Smith-Waterman algorithm. This alignment scoring is based on sequence complementarity and not on sequence identity. In other words, it looks for A:U and G:C matches instead of A:A, G:G, etc. The G:U wobble pair is also permitted which are important for the accurate detection of RNA:RNA duplexes. In the second phase, the algorithm takes high scoring alignment, detected from phase 1 and estimates the thermodynamic stability of RNA duplexes based on these alignments. This second phase of the method utilizes folding routines from the RNAlib library, which is a part of the ViennaRNA package.

All the filtered pairs from the modified miRanda were considered as most likely to be true miRNA-target pairs. The position-specific nature of miRNAs binding to corresponding gene targets motivated us to design a tool which can give details of position specific targets too.

The efficacy of predicted miRNA interaction is measured by parameters such as maximum sequence complementarity with target genes, query length and thermodynamically minimum hybridization energy between a miRNA and its target RNA. Position specific and positive results were extracted from miRanda outputs using an in-house Linux shell script. PAmiRDB entries are presented in a tab-separated format using a PERL script, combining information from the Genbank regarding target site along with target genes details.

PAmirDB database is implemented as a MySQL database, connected through PHP scripts to dynamically generate user-friendly HTML output for user-defined customized queries, supported by an Apache web server.
